# Enzymatic Strategies and Carbon Use Efficiency of a Litter-Decomposing Fungus Grown on Maize Leaves, Stems, and Roots

**DOI:** 10.3389/fmicb.2016.01315

**Published:** 2016-08-26

**Authors:** Gwenaëlle Lashermes, Angélique Gainvors-Claisse, Sylvie Recous, Isabelle Bertrand

**Affiliations:** ^1^INRA, UMR614 Fractionnement des AgroRessources et EnvironnementReims, France; ^2^Université Reims-Champagne Ardenne, UMR614 Fractionnement des AgroRessources et EnvironnementReims, France; ^3^INRA, UMR1222 Eco&SolsMontpellier, France

**Keywords:** carbon-use efficiency, lignocellulose decomposition, extracellular enzymes, fungi, litter quality, soil, enzyme efficiency

## Abstract

Soil microorganisms can control the soil cycles of carbon (C), and depending on their C-use efficiency (CUE), these microorganisms either contribute to C stabilization in soil or produce CO_2_ when decomposing organic matter. However, little is known regarding the enzyme investment of microbial decomposers and the effects on their CUE. Our objective was to elucidate the strategies of litter-decomposing fungi as a function of litter quality. Fungal biosynthesis and respiration were accounted for by quantifying the investment in enzyme synthesis and enzyme efficiency. The basidiomycete *Phanerochaete chrysosporium* was grown on the leaves, stems, and roots of maize over 126 days in controlled conditions. We periodically measured the fungal biomass, enzyme activity, and chemical composition of the remaining litter and continuously measured the evolved C–CO_2_. The CUE observed for the maize litter was highest in the leaves (0.63), intermediate in the roots (0.40), and lowest in the stems (0.38). However, the enzyme efficiency and investment in enzyme synthesis did not follow the same pattern. The amount of litter C decomposed per mole of C-acquiring hydrolase activity was 354 μg C in the leaves, 246 μg C in the roots, and 1541 μg C in the stems (enzyme efficiency: stems > leaves > roots). The fungus exhibited the highest investment in C-acquiring enzyme when grown on the roots and produced 40–80% less enzyme activity when grown on the stems and leaves (investment in enzymes: roots > leaves > stems). The CUE was dependent on the initial availability and replenishment of the soluble substrate fraction with the degradation products. The production of these compounds was either limited because of the low enzyme efficiency, which occurred in the roots, or because of the low investments in enzyme synthesis, which occurred in the stems. Fungal biosynthesis relied on the ability of the fungus to invest in enzyme synthesis and the efficient interactions between the enzymes and the substrate. The investment decreased when N was limited, whereas the efficiency of the C-acquiring enzymes was primarily explained by the hemicellulose content and its embedment in recalcitrant lignin linkages. Our results are crucial for modeling microbial allocation strategies.

## Introduction

There has been a growing interest over the past decade in reaching a more comprehensive understanding of microbial behavior in relation to soil processes because of the importance of microbial communities in controlling the soil cycles of carbon (C) and nutrients, such as nitrogen (N), as well as their potential feedback under changing environments (Wieder et al., 2013). Substantial efforts have been made toward the proposal of new conceptual frameworks that more explicitly describe the interactions between microorganisms and their substrates during plant litter decomposition (e.g., Allison, 2012; Cotrufo et al., 2013). Several functional groups of microorganisms have been described and differentiated according to their physiological characteristics and enzymatic activities. As an example of this approach, Moorhead and Sinsabaugh (2006) used a guild-based model to simulate the decomposition of litters in soil by three microbial guilds: (i) “opportunists,” which grow rapidly and consume soluble substrates, (ii) “decomposer specialists,” which grow more slowly and consume cellulose and lignocellulose, and (iii) “miners,” which grow very slowly and are specialized in lignin degradation. The physiological and enzymatic traits are key for partitioning C mineralization and C stabilization in soil, and microbial residues are thought to contribute strongly to stabilization (Miltner et al., 2012; Clemmensen et al., 2013; Cotrufo et al., 2015). Microbial C-use efficiency (CUE) is one of the main integrative descriptors of this partitioning, and it has recently received much attention (Manzoni et al., 2012; Sinsabaugh et al., 2013; Allison, 2014; Geyer et al., 2016). Microbial CUE is defined here as the C retained as new microbial biomass divided by the total C taken up from the substrate, with the difference primarily attributed to respiration.

Another important feature of biogeochemical processes concerns the interactions among C and N cycles, which govern the allocation of microbial resources toward C or N acquisition through the synthesis of extracellular enzymes (Sinsabaugh et al., 2008). A series of different enzymes are required to degrade complex organic substrates, including substrate-specific hydrolases that target C or nutrients and non-specific oxidative enzymes, such as laccase (Schimel and Schaeffer, 2012). Microorganisms direct their resources toward these different classes of enzymes to receive available forms of C and nutrients that match their stoichiometric needs (Sinsabaugh et al., 2008). This microbial resource allocation is also constrained by the cellular metabolic abilities (Molenaar et al., 2009), the resources available for enzyme synthesis (Mooshammer et al., 2014), or their substrate decomposition strategies (Osono, 2010). Indeed, once enzymes have been secreted, their activities have shown different efficiencies, meaning that the amount of enzyme activity required for substrate decomposition can vary markedly. High contents of lignin may decrease enzyme efficiency (Sinsabaugh et al., 2002; Wickings et al., 2012), and recalcitrant substrates may alternatively induce the synthesis of an enzyme “cocktail” in which hydrolytic enzymes act in synergy with oxidative enzymes, thereby resulting in a higher efficiency relative to enzymes secreted on labile substrates (Amin et al., 2014). Enzyme activities per unit microbial biomass synthetized over a specific period are used to characterize the microbial “investment” in enzymes. A strong investment into enzyme activities should incur an extra respiratory cost to microbes to support enzyme synthesis (Moorhead et al., 2012; Cotrufo et al., 2013). This extra cost may reduce the CUE or be compensated for by higher biosynthesis by using the available compounds produced by the enzymes (and thus depending on their efficiency). Observations of the metabolic and enzymatic responses reported in soil decomposition studies are usually reported as the mean responses of the microbial communities. At the population scale, the CUE is usually determined with chemostats, for which the optimal growth conditions are used to estimate the CUE near the theoretical maxima because they are only constrained on simple substrates by the thermodynamic and intrinsic metabolic limits of the population (Geyer et al., 2016). Although determining the CUE of individual microbial species is necessary, it is not feasible to perform *in situ*, which was recently highlighted by Geyer et al. (2016). Some studies have been conducted on individual species growing on plant litters, but these did not report the microbial biosynthesis and respiration data required to calculate CUE or did not explicitly calculate this parameter (e.g., Boberg et al., 2008; Allison et al., 2009; Song et al., 2012). These laboratory microcosm approaches with individual species can capture the drivers of the microbial metabolism of a population, including growth respiration and cellular maintenance, as proposed by Geyer et al. (2016). However, we believe that these approaches also have the potential to provide some insights regarding the community and ecosystem temporal and ecological scales. Drivers at the community scale include substrate stoichiometry and the sensitivity of microorganisms to environmental conditions, and drivers at the ecosystem scale can include interactions with the environmental matrix and biomass turnover.

Therefore, we aimed to explicitly describe the first determination of the CUE for an individual species population during growth on plant litters of varied biodegradability, taking into account the effect of the bioavailability of the substrate C and N as additional “community drivers” that affect the CUE. Specifically, we aimed to examine whether the microorganisms’ enzymatic strategies, i.e., their investment in extracellular enzyme activity and the efficiency of these activities, modified their CUE. In this study, we analyzed the behavior of a litter-decomposing fungus, the saprotrophic basidiomycete *Phanerochaete chrysosporium*. This fungus is known to be able to completely degrade all major components of plant cell walls, i.e., cellulose, hemicellulose, and lignin (Kersten and Cullen, 2007; Ray et al., 2012). In the absence of competitors, we hypothesized that *P. chrysosporium* would secrete a wide range of extracellular hydrolytic and oxidative enzymes and behave as a “decomposer specialist” (Moorhead and Sinsabaugh, 2006). Using a model microorganism growing on a substrate without soil made it possible to determine an unambiguous link between the microbial C metabolism (biomass production and respiration) and the activities and efficiencies of the enzymes produced during litter degradation. As the substrate models, we selected three litters that are known to exhibit varied intrinsic degradabilities: maize leaves, which is a labile substrate rich in low-lignified primary cell walls, and maize stems and roots, which are recalcitrant substrates rich in secondary cell walls with lignin and embedded polysaccharides in lignin (Amin et al., 2014). We hypothesized that the fungus will adapt the synthesis of its various enzymes of different classes to the chemistry of the substrate toward the acquisition of the required nutrient. We also hypothesized that investment in enzymes will be a priority for the fungus and that the enzyme efficiencies will thus mainly govern the CUE. Greater enzyme efficiencies will increase the amount of available compounds used in the production of new fungal biomass, increasing the CUE.

## Materials and Methods

### Experimental Design and Samplings

Maize (*Zea mays* L.) shoots and roots were harvested at the plant’s physiological maturity in the field. We removed leaves from the stover, selected stem internodes 5–9 (counting from the bottom) and roots of 2–3 mm in diameter. The roots were washed with 50 g L^-1^ sodium meta-phosphate solution for 24 h and rinsed with deionized water to remove any soil particles. All of the litter samples were dried for 1 week at 35°C and then cut into fragments approximately 1 cm wide before sterilization with two cycles of γ-irradiation at an intensity of 45 KGy (Ionisos, Dagneux, France).

*Phanerochaete chrysosporium* BRFM531 was obtained from the International Centre of Microbial Resources (INRA, Marseille, France). The fungus was grown in the dark at 25°C in potato-dextrose agar medium (0.4% potato, 2% glucose, 0.1% agar). Spores of the 1-week-old fungal culture were scraped into sterile distilled water, and this spore suspension was used as the inoculum.

Litter samples of 5 g dry matter (DM) were either inoculated with 2.4 × 10^7^ spores g^-1^ DM or not inoculated at all (control litter). We adjusted the litter moisture with sterile water to 75–80% of wet weight to avoid moisture growth limitations. The inoculated and control litters were incubated in 1200-mL sterile hermetic jars at 25°C for 126 days in the dark. One or two vials containing 10 mL of 1 M sodium hydroxide were placed in each jar along with another vial containing 10 mL of sterile water to maintain a constant relative humidity. We periodically opened all of the microcosms under aseptic conditions to maintain aerobic conditions and replace the sodium hydroxide traps. The concentration of trapped CO_2_ was measured by continuous flow colorimetry using an auto-analyzer (TRAACS 200, Bran and Luebbe, Norderstedt, Germany). Three microcosms per inoculated litter type and two control litters were collected at the beginning of the experiment and after 14, 28, 56, and 126 days of incubation for the litter chemistry analysis, enzyme assays and fungal biomass quantification.

### Litter Chemistry

The inoculated and control litters were lyophilized and ground to 80 μm to determine the total C and N contents by elemental analysis (NA 2000, Fisons Instruments, Milan, Italy), with the dissolved organic C (DOC) and N (DON) determined by a water extraction (water:litter, 10:1, v/w, 30 min, 20°C). The DOC contents of the extracts were analyzed using an auto-analyzer (1010, O.I. Analytical Aurora Model 1030), and the DON was calculated as the difference in the total N and NO_3_^-^ content. The total soluble N content was determined by dry combustion according to the standard ISO 13878 (AFNOR, 1998), and the NO_3_^-^ content was measured by continuous flow colorimetry. The soluble compounds (SOL) were extracted from 1 mm ground litter samples in hot water (30 min, 100°C) and then in neutral detergent (1 h, 100°C; Van Soest and Wine, 1967). The resulting residues were the cell-wall fractions, and these were dried at 37°C and ground to 80 μm. The residual moisture content of the cell-wall fractions was measured for separate samples at 80°C and considered in the calculations. An analysis of the chemical composition was performed as described previously (Bertrand et al., 2006). Briefly, the cell-wall sugars were extracted using a two-step hydrolysis (10 mg cell-wall residue, 125 μL 12 M sulfuric acid for 2 h at 20°C and then 1 M sulfuric acid for 2 at 100°C; Blakeney et al., 1983). The released saccharides were separated by high performance anion-exchange chromatography (HPAEC) on a CarboPac PA-1 column (4 mm × 250 mm, Dionex, Thermo Fisher Scientific, USA). The monosaccharide composition was analyzed and seven sugars were quantified using 2-deoxy-D-ribose as an internal standard along with standard solutions of the following neutral carbohydrates: L-arabinose (Ara), D-glucose (Glu), D-xylose (Xyl), D-galactose (Gal), D-mannose, D-rhamnose, D-galacturonic acid, and D-glucuronic (Beaugrand et al., 2004). The Klason lignin (KL) content was approximated as the acid-unhydrolyzable residue remaining after a two-step acid hydrolysis of the cell-wall polysaccharides (300 mg cell wall residue, 3 mL 12 M sulfuric acid for 2 h at 20°C and then 1 M sulfuric acid for 2 h at 100°C), correcting for the ash measurement (Monties, 1984).

The chemical composition of the selected litters was compared (**Table 1**), and the highest SOL and DOC contents were observed in the leaves, higher KL contents were observed in the stems and roots than in the leaves, and the lowest N and DON contents were observed in the stems. Glu was used as a proxy for cellulose, and the sum of all of the sugars except Glu was used as a proxy for hemicellulose (Bertrand et al., 2006). The ratio of Ara:Xyl was used to assess the level of hemicellulose ramifications (Amin et al., 2014).

**Table 1:** Initial chemical composition of the maize litters (mean ± standard error).

% Dry matter	Leaves	Stems	Roots	*P*-value
Total C	47.3 ± 0.4^a^	48.6 ± 0.2^a^	47.8 ± 0.1^a^	0.099
Total N	1.66 ± 0.02^a^	0.30 ± 0.03^c^	0.86 ± 0.1^b^	0.001^∗∗^
C to N ratio	28.5 ± 0.2^b^	162.6 ± 18.7^a^	56.2 ± 8.1^b^	0.018^∗^
Dissolved organic C (DOC)	6.9 ± 0.4^a^	5.0 ± 0.8^a^	3.6 ± 0.2^a^	0.101
Dissolved organic N (DON)	0.31 ± 0.00^a^	0.07 ± 0.02^b^	0.12 ± 0.00^b^	0.006^∗^
DOC to DON ratio	22.2 ± 0.8^c^	67.8 ± 5.8^a^	30.1 ± 0.3^b^	0.001^∗∗^
Soluble fraction	29.7 ± 0.3^a^	18.5 ± 1.3^b^	13.1 ± 2.3^b^	0.009^∗^
Cell Wall to N ratio	42.3 ± 0.7^b^	273.2 ± 34.8^a^	101.9 ± 11.7^b^	0.028^∗^
Total cell wall sugars	43.6 ± 1.2^a^	57.1 ± 2.0^a^	58.9 ± 2.4^a^	0.050
D-Glucose	24.3 ± 0.6^a^	36.5 ± 1.3^a^	36.6 ± 1.7^a^	0.040
D-Xylose (Xyl)	15.0 ± 0.7^a^	18.0 ± 0.5^a^	18.0 ± 0.6^a^	0.060
L-Arabinose (Ara)	2.5 ± 0.1^a^	1.7 ± 0.1^b^	2.8 ± 0.1^a^	0.011^∗^
D-Galactose	1.0 ± 0.3^a^	0.4 ± 0.0^a^	1.2 ± 0.1^a^	0.164
Ara to Xyl ratio	0.16 ± 0.00^a^	0.09 ± 0.00^c^	0.15 ± 0.00^b^	0.000^∗∗^
Klason lignin (KL)	11.7 ± 0.2^b^	15.7 ± 0.1^a^	16.6 ± 0.7^a^	0.021^∗^
KL to N ratio	7.0 ± 0.0^c^	52.5 ± 5.6^a^	19.4 ± 1.9^b^	0.003^∗^
Lignocellulose index^d^	0.21 ± 0.00^b^	0.22 ± 0.01^a^	0.22 ± 0.00^a^	0.026^∗^


*Significant differences among the residues were tested using a one-way ANOVA (*n* = 6, significance levels ^∗^*P* > 0.05, ^∗∗^*P* > 0.01). Different letters within rows indicate significant differences among the residues (Tukey’s HSD test, α = 0.05).*

*^d^Ratio of Klason lignin to (Klason lignin + Total cell-wall sugars).*

### Enzyme Assays

We determined the activity of six enzymes: endo-1,4-β-glucanase (EG, which hydrolyzes glycosidic internal linkages of cellulose; EC 3.2.1.4), endo-1,4-β-xylanase (EX, which hydrolyzes internal linkages of xylan; EC 3.2.1.8), β-1,4-glucosidase (βG, which hydrolyzes cellobiose into glucose; EC 3.2.1.21), laccase (LAC, which oxidizes phenols; EC 1.10.3.2), L-leucine aminopeptidase (LAP, which liberates leucine and other amino acids by protein hydrolysis; EC 3.4.11.1), and *N*-acetyl-glucosaminidase (NAG, which hydrolyzes chitin into *N*-acetyl-glucosamine; EC 3.2.1.50). For each treatment, 2.2 g litter DM was placed into 100 mL of 50 mM sodium acetate buffer at pH 5 and blended with a Waring blender at 4°C for 60 s for the maize leaves and stems and for 2 min for the roots (Bell et al., 2013). The suspensions were then vacuum filtered through 0.45 μm nylon filters followed by 1.2 μm glass-fiber filters and frozen prior to the enzyme assays by colorimetry and fluorometry.

#### Colorimetric Assays

We measured the EG and EX activities using AZO-CM-Cellulose (Megazyme) and Remazol Brilliant Blue R D-xylan (Fluka) as substrates, respectively, as described previously (Amin et al., 2014). For each treatment, the enzyme activities were measured in triplicate by adding 0.5 mL of the litter extracts to 0.5 mL of the substrate solution. Once the reaction mixtures had been incubated for 3 h at 25°C and pH 5, the EX reactions were stopped by adding 2 mL of ethanol (96%, v/v) followed by 10 min of vortexing and the EG reactions were stopped by adding 2 mL of hydroalcoholic solution (76%, v/v) of sodium acetate trihydrate (294 mM) and zinc acetate (21.8 mM). The absorbance of the supernatants was measured at 590 nm using a spectrophotometer (Heλos γ, Thermo Spectronic, UK). We determined the EG and EX activities for all of the samples by referencing a standard curve obtained with commercial EG from *Aspergillus niger* (Megazyme) and purified EX from *Thermobacillus xylanilyticus*.

The activity of βG was assayed in microplates using *p*-nitrophenol-β-D-glucoside (PNPG) in seven replicates. The reaction mixtures contained 60 μL of litter extract and 60 μL of a 5 mM PNPG solution in 50 mM sodium acetate buffer, and they were incubated at 50°C and pH 5 for 10 min as described by Norkrans (1957). The reactions were stopped by adding 120 μL of 1 M sodium carbonate solution, and the absorbance was measured at 400 nm using a microplate spectrophotometer (VersaMax, Molecular Devices Corporation, Sunnyvale, CA, USA). We determined the βG activity by referencing a standard curve obtained from a commercial βG from almonds (Megazyme).

We measured the LAC activity by monitoring the oxidation of ABTS (2,2′-azino-bis-3-ethylbenzothiazoline-6-sulfonic acid diammonium salt, Sigma–Aldrich) in sodium acetate buffer on microplates as described by Floch et al. (2007). Each treatment was measured in eight replicates by adding 200 μL of the litter extracts to 40 μL of the substrate solution. After incubating the reaction mixtures for 10 min at 25°C and pH 4, the absorbance of the supernatant solution was measured at 414 nm using a microplate spectrophotometer (VersaMax). We quantified the LAC activity with respect to ABTS degradation using a commercial LAC from *Trametes versicolor* (Sigma–Aldrich).

#### Fluorimetric Assays

The substrates 7-amino-4-methyl coumarin hydrochloride (7-AMC-leucine), which was used to measure LAP activity, and 4-methylumbelliferyl-β-D-glucosaminidine (4-MUB-NAG), which was used to measure NAG, as well as their respective fluorogenic compounds (4-MUB and 7-AMC) were obtained in crystalline form from Sigma–Aldrich. For each treatment, the activities were assayed in six replicates using black microplates by adding 50 μL of the 200 μM substrate solution and 200 μL of the litter extract diluted in acetate buffer. This solution was incubated at room temperature for 3 h for the LAP assays and for 30 min for the NAG assays at pH 6. The reactions were terminated by adding between 8 and 20 μL of 1 M sodium hydroxide solution to raise the pH to 9 and optimize the fluorescence (Bell et al., 2013). After excitation at 365 nm, the fluorescence emission intensity of the sample was measured at 460 nm using a microplate fluorimeter (Spectramax Gemini, Molecular Devices Corporation, Sunnyvale, CA, USA) and corrected for quenching as described in German et al. (2011). We determined the LAP activity by referencing a standard curve obtained from commercial LAP from porcine kidney (Sigma–Aldrich). A standard curve of the fluorescence intensities for the specific fluorogenic product (4-MUB) of the substrate (4-MUB-NAG) was used to quantify the NAG activity.

### Quantification of Fungal Biomass

We used ergosterol as an indicator of the living fungal biomass (e.g., Montgomery et al., 2000). Whole samples of fungal cultures on litter were lyophilized and ground to 80 μm according to the method of Gessner and Schmitt (1996). Once the samples (100 mg) were extracted with 5 mL of a methanol solution with 8 g L^-1^ of potassium hydroxide for 30 min at 80°C, they were cooled in the dark before the addition of 1 mL of neutralization solution (hydrochloric acid, 0.65 M). These extracts (3 mL) were purified with a solid phase extraction (SPE) by the addition of 1 mL of pure methanol solution using hydrophilic-lipophilic cartridges (Waters Oasis HLB 60 mg 3 cc). The ergosterol was eluted four times with a solution of isopropanol with potassium hydroxide and methanol. The extracts were filtered through a 0.45 μm polytetrafluoroethylene (PTFE) filter and frozen before analysis. The ergosterol content was quantified using high-performance liquid chromatography (HPLC, Waters, USA) on a C18 reversed-phase column at 30°C (Waters Spherisorb ODS-2–5 μm, 250 mm × 4.6 mm), with methanol as the mobile phase at a flow rate of 1 mL min^-1^ and UV detection at 282 nm.

To determine the relationship between the ergosterol content and the fungal biomass, we also cultivated *P. chrysosporium* BRFM531 in liquid medium MA2 (malt 2%) in the dark at 25°C and quantified the dry mass of the fungal mycelium and its ergosterol content in two replicates. Because the ergosterol content of the fungal mycelia did not change significantly with time (*P* < 0.001; 1618 ± 524, 1328 ± 466, 1361 ± 153, 1337 ± 246 μg g^-1^ of dry mycelia after 7, 14, 21, 28 days, respectively), the mean value of 1411 ± 139 μg ergosterol g^-1^ of dry *P. chrysosporium* mycelium was used as a conversion factor for the calculations.

We collected the mycelium of *P. chrysosporium* at its maximum biomass production stage in a preliminary experiment, and the fungal C and N contents that were measured by the elemental analysis (NA 2000, Fisons Instruments, Milan, Italy) were 46.6 ± 3.2% DM and 6.5 ± 0.2% DM, respectively.

### Data Treatment

The metabolic responses of *P. chrysosporium* in the cultures on the maize litter were first assessed by calculating the fungal CUE (Keiblinger et al., 2010; Manzoni et al., 2012) between days 0 and 14 of incubation using the following equation:

$$CUE=\frac{\Delta C_{f}}{\Delta C_{f}+\Sigma {CO}_{2}}$$

where ΔC_f_ is the increase in fungal biomass (in mg fungal C g^-1^ initial litter C) and ΣCO_2_ is the cumulative CO_2_ respired at day 14 (in mg C-CO_2_ g^-1^ initial litter C). Next, we calculated the metabolic quotient (*q*CO_2_), which is defined as the C respired per unit microbial biomass C (Spohn, 2015) and expressed in μg C-CO_2_ mg^-1^ fungal C day^-1^, using the following equation:

$$q{CO}_{2}=\frac{\Sigma {CO}_{2}*1000}{14*\Delta C_{f}}$$

The enzyme activities were expressed as the micromoles of reaction product per min per gram of initial DM of litter. The enzyme activity (μmol min^-1^ g^-1^ DM) values reported for the control litters were low and did not vary with the incubation time. These values were subtracted from their corresponding inoculated treatments to focus on the fungal activities. Specific enzyme activities were calculated by dividing the enzyme activities by the fungal biomass (in μmol min^-1^ mg^-1^ fungal C). Hereafter, the abbreviations EGs, EXs, βGs, LAPs, NAGs, and LACs denote the specific enzyme activities.

The cumulative enzyme activities per unit fungal C were defined as the fungal investment in enzyme activities. The cumulative enzyme activity (in μmol mg^-1^ fungal C) was calculated by multiplying the average specific enzyme activity between two dates by the time span and then summing these activities over the entire incubation period (Wickings et al., 2012; Moorhead et al., 2013b). C-acquiring hydrolase activity was assessed by summing the βG, EX and EG activities, N-acquiring hydrolase activity was assessed by summing the LAP and NAG activities, and oxidative activity was assessed using the LAC value. We standardized this investment to a 0–100% scale by dividing each value by the maximum value among the litter treatments. We calculated the enzyme efficiencies between dates and summed the values over the entire incubation period for the C-acquiring hydrolase activity by summing the βG, EX, and EG activities and for the oxidative enzyme activity by using the LAC value. The enzyme activity efficiencies can be assessed against the CO_2_ mineralized as an index of microbial metabolism (Sinsabaugh et al., 2002; Moorhead et al., 2013b; Amin et al., 2014). We proposed in this study to calculate the efficiency of the enzyme activity for fungal-C biosynthesis (in μg fungal-C mol^-1^) by dividing the amount of recovered C in fungal biomass by the cumulative enzyme activity (in μmol g^-1^ DM). We also assessed the enzyme efficiency for C decomposition by dividing the cumulative decomposed C (i.e., the sum of the respired C and recovered C in the fungal biomass) by the cumulative enzyme activity (in μg decomposed-C mol^-1^).

Ratios of the natural logarithms of (βG + EX + EG) activity to the natural logarithms of (LAP + NAG) activity were calculated to analyze the fungal balance in activities of the extracellular enzymes responsible for C and N acquisitions as first proposed by Sinsabaugh et al. (2008). These ratios are often used as a proxy for the relative overinvestment in enzymes by targeting a limiting element to the microorganisms (Moorhead et al., 2013b).

### Statistics

The presented results are the means and standard deviations of the replicated microcosms for each litter treatment. The litter chemical characteristics, enzyme efficiencies, and enzyme activity ratios were compared across litter treatments using a one-way ANOVA and Tukey’s HSD (α = 0.05) tests. To avoid false discovery related to multiple testing, we corrected all of the *P*-values using the Benjamini and Hochberg false discovery rate control (Verhoeven et al., 2005). Stepwise regressions were performed using the software Sigmaplot (Version 12.0, Systat Software, Inc., Germany) to identify the best model for describing the relationships between the significant litter chemical characteristics and metabolic responses of *P. chrysosporium* growing on these litters (CUE, ΣCO_2_, C_f_ at day 14).

A multivariate redundancy analysis (RDA) was performed using the software XLSTAT-ADA (version 2015.2, Addinsoft, Paris, France) to extract and summarize the variation in a set of metabolic and enzymatic response variables (of *P. chrysosporium* growing on maize litter) that can be explained by a set of explanatory variables for the litter chemical composition (Buttigieg and Ramette, 2014). All variables were center-reduced to perform the analysis. We used the main chemical characteristics as the independent variables, and we used the cumulative mineralized and recovered C in the fungus (in mg C g^-1^ initial litter C), βGs, EGs, EXs, LAPs, NAGs, LACs activity (in μmol min^-1^ mg^-1^ fungal C), natural logarithms ratio of (βG + EX + EG) activity versus (LAP + NAG) activity (in μmol min^-1^ g^-1^ DM), and enzymatic C decomposition efficiency for βG activity (in μg decomposed C mol^-1^) as the response variables. All of the data were checked for normality and transformed to meet the requirements before the analysis.

## Results

### Metabolic Responses of *P. chrysosporium*

**FIGURE 1:**
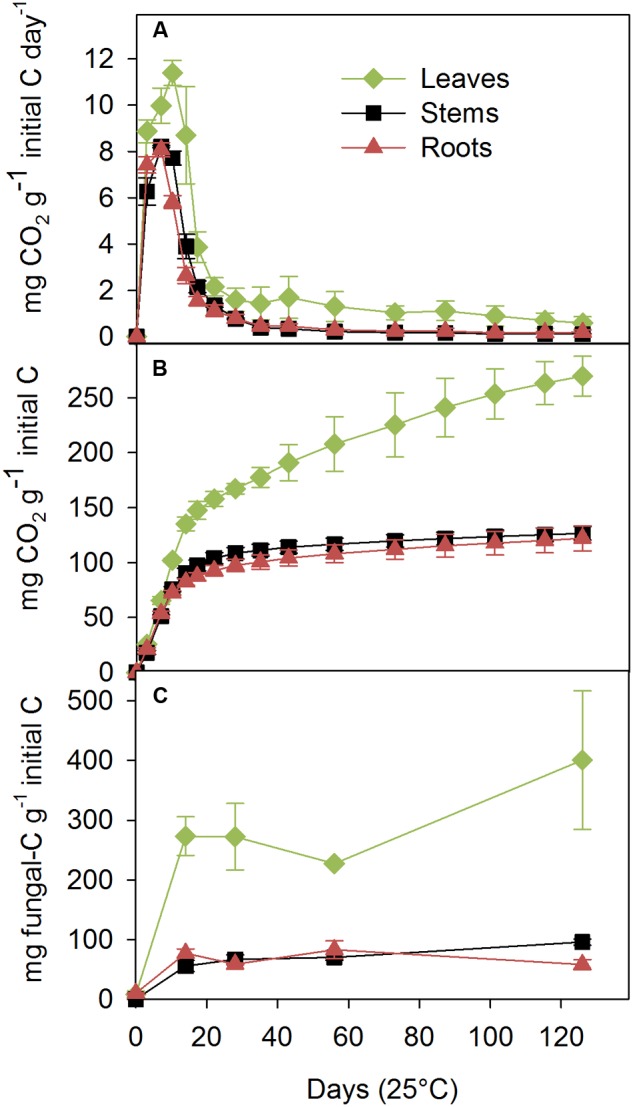
**Carbon mineralization rates **(A)**, cumulative C mineralized **(B)** and fungal C dynamics **(C)** measured during a 126-day incubation at 25°C with the saprotrophic basidiomycete *Phanerochaete chrysosporium* on maize leaf, stem and root litters.** The results are expressed per gram of initial (non-decomposed) organic C content of the litter. The values represent the means of three replicated microcosms ± SE.

*Phanerochaete chrysosporium* fully colonized the three inoculated litters, whereas the control litters were not colonized by any microorganism throughout the experiment. C–CO_2_ was not emitted by the control litter. In the inoculated treatments, the rate of C mineralization was the highest for the leaves, peaked at 11.4 ± 0.5 mg C–CO_2_ g^-1^ initial litter C day^-1^ at day 10, and then rapidly decreased to a second lower peak of 1.7 ± 0.9 mg C–CO_2_ g^-1^ initial litter C day^-1^ on day 43 (**Figure 1A**). The C mineralization rates for the stems and roots were similar but lower than that for leaves, and they presented a single peak at day 7 of 8.2 ± 0.0 and 8.1 ± 0.3 mg C–CO_2_ g^-1^ initial litter C day^-1^, respectively. The cumulative C mineralized over 126 days was highest in the leaves, intermediate in the stems, and lowest in the roots (270 ± 18, 127 ± 6, and 124 ± 11 mg C–CO_2_ g^-1^ initial litter C, respectively; **Figure 1B**), which indicated that 27% (leaves), 13% (stems), and 12% (roots) of the added C had been mineralized.

The ergosterol concentration after 14 days ranged from 419 ± 50 μg g^-1^ substrate DM for the leaves, 84 ± 14 for the stems, with the roots presenting an intermediate value of 118 ± 11 μg g^-1^ DM (data not shown). Although, the fungal biomass increased throughout the entire experiment on the leaf and stem substrates, the most significant fungal production was observed during the first 14 days. The C accumulation in the fungus was estimated using the conversion factor from ergosterol-to-fungal biomass and fungal C concentration (**Figure 1C**). At the end of the experiment, 40% ± 12, 10% ± 1, and 6% ± 1 of the initial litter C was recovered as fungal C in the leaf, stem and root treatments, respectively.

After 14 days, *P. chrysosporium* had accumulated 129 ± 15 (leaves), 27 ± 4 (stems), and 38 ± 4 (roots) mg C g^-1^ initial litter DM in its biomass (data not shown). Assuming strict homeostasis and a negligible adjustment for extracellular enzyme synthesis and using the measured C:N ratio of *P. chrysosporium* (7.1), the N accumulated in the fungus was 18.2 ± 2.2 (leaves), 3.8 ± 0.6 (stems), 5.3 ± 0.5 (roots) mg N g^-1^ initial litter DM over the same period. These amounts of fungal N are closed to the total initial litter N (16.6 in the leaves 3.0 in the stems, 8.6 mg N g^-1^ initial litter DM in the roots, **Table 1**).

**FIGURE 2:**
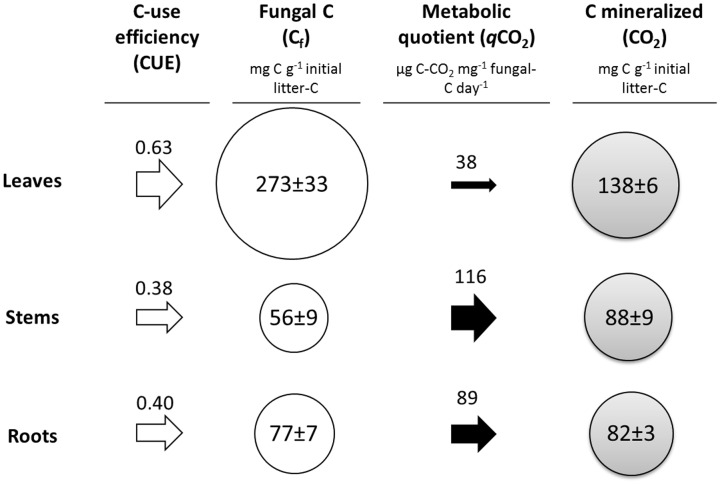
**Schematic representation of the metabolic responses of *P. chrysosporium* in culture on maize leaf, stem, and root litters after 14 days.** Circles represent the proportional amount of C accumulated in the fungal biomass (white) and C mineralized (gray). The arrows represent the C-use efficiency (white) and metabolic quotient (black). The values represent the means of three replicated microcosms ± SE.

The litter type had a strong effect on the metabolic response of *P. chrysosporium* during substrate degradation (**Figure 2**). The high CUE obtained for the leaves (0.63) reflects the strong ability of the fungus to use C from this litter in biosynthesis. However, because of the high simultaneous mineralization rate, the fungal metabolic quotient (*q*CO_2_) was low (38 μg C–CO_2_ mg^-1^ fungal C day^-1^) compared with the two other substrates. Conversely, *P. chrysosporium* incorporated less C per gram of initial litter C when grown on the stems (CUE = 0.38), whereas its microbial metabolism was more active and resulted in a *q*CO_2_ value of 116 μg C–CO_2_ mg^-1^ fungal C day^-1^. The fungus showed intermediary metabolic responses for the roots as indicated by the CUE (0.40) and *q*CO_2_ (83 μg C–CO_2_ mg^-1^ fungal C day^-1^) measurements.

### Enzymatic Responses of *P. chrysosporium*

**FIGURE 3:**
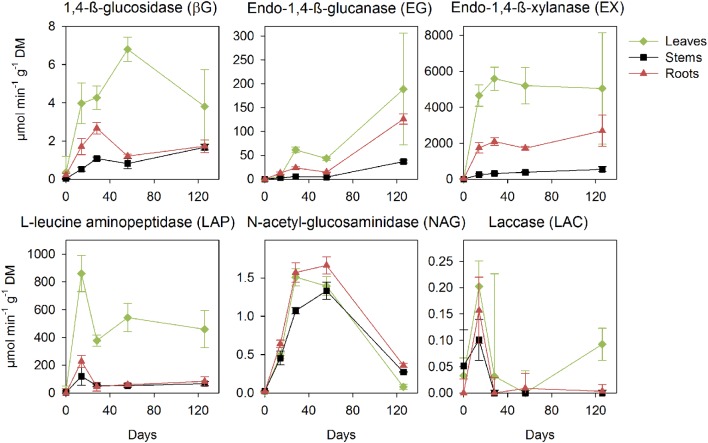
**Potential activities of the extracellular enzymes (in μmol min^-1^ g^-1^ litter dry matter) during decomposition of the maize leaf, stem and root litters by *P. chrysosporium* measured during a 126-day incubation.** The values represent the means of three replicated microcosms ± SE.

*Phanerochaete chrysosporium* produced hydrolase activities early in decomposition concomitantly with biomass production for the C-acquisition (βG and EX) and N-acquisition (LAP and NAG) enzymes (**Figure 3**). The litters induced differences in the magnitude of activity, with the highest peak activities observed in the leaves, intermediary activities detected in the roots and the lowest activities found in the stems for all enzymes. The activities for βG, EX, and LAP increased from the early phase of decomposition, and the same was found for LAC, which presented a marked peak on day 14. The activities of the endo-cleaving enzyme EG started after the fungal production phase and peaked at the end of the incubation. NAG activity peaked after the fungal production phase, and this was followed by a marked decrease at the end of the experiment.

**FIGURE 4:**
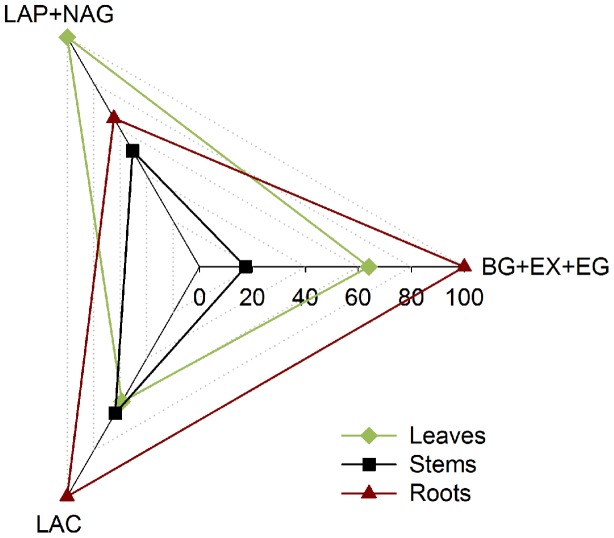
**Relative synthesis of the cumulative enzyme activities per unit of fungal C characterizing the investment of *P. chrysosporium* in the enzymes activities for C-hydrolase (βG + EX + EG), N-hydrolase (LAP + NAG), and oxidative enzymes (LAC) during the decomposition of maize leaves, stems and roots and measured over a 126-day period**.

The fungal investments in enzyme activities showed marked differences in the intensity and allocation of microbial resources toward the different classes of enzymes on the different litters (**Figure 4**). We observed increased enzyme activity per unit of fungal biomass when *P. chrysosporium* was grown on the roots for the C-acquiring hydrolase (βG + EX + EG) and oxidative enzymes (LAC), and approximately 40% less enzyme activity was produced per unit of fungal C on the leaves and 40–80% less was produced on the stems. However, the fungus invested approximately 40–50% more in the N-acquiring enzymes (LAP + NAG) when grown on the leaves compared with the stems and roots.

**FIGURE 5:**
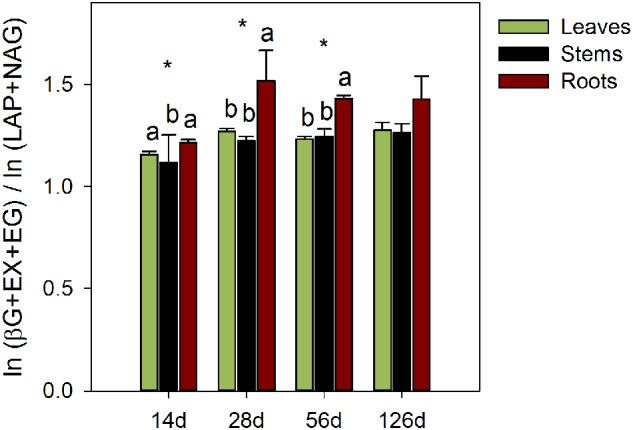
**Ratio of the enzymatic C to N acquisition activities during the decomposition of maize leaf, stem and root litters by *P. chrysosporium* measured after 14, 28, 56, and 126 days.** The values represent the means of three replicated microcosms ± SE. Significant differences among litters were tested using a one-way ANOVA (*n* = 9; significant level: ^∗^*P* < 0.05; Benjamini and Hochberg correction for *P*-values). Different letters indicate significant differences among litters at each date (Tukey’s HSD test, α = 0.5).

The efficiencies of the C-acquiring hydrolase activity were significantly higher in the stem litter than in other litters, for fungal-C accumulation (*P* < 0.01) and C decomposition (*P* < 0.01; **Table 2**). In contrast, the efficiencies of the oxidative enzymes among the litter treatments were not significantly different. The amounts of litter C mineralized and recovered in the fungal biomass per unit of C-acquiring hydrolase activity were the highest on the stems. The ratios of the enzyme activities produced by *P. chrysosporium* toward C or N acquisition showed significant relative overinvestments (*P* < 0.05) in N-acquiring enzymes on the stems after 14 days, which indicated a N limitation, and in C-acquiring enzymes on the roots after 28 and 56 days, which indicated a C limitation (**Figure 5**).

**Table 2:** Enzymatic efficiency of the C-acquiring enzymes (mean ± SE) for fungal-C accumulation and C decomposition during the decomposition of the maize litters by *P. chrysosporium.*

	Unit	Leaves	Stems	Roots	*P*-value
**Fungal-C accumulation**
βG+EX+EG	μg fungal-C mol^-1^	222 ± 87^b^	678 ± 18^a^	82 ± 8^c^	0.000^∗∗∗^
LAC	pg fungal-C mol^-1^	20 ± 6^a^	21 ± 10^a^	7 ± 2^a^	0.068
**C decomposition (CO_2_ mineralization + fungal-C accumulation)**	
βG+EX+EG	μg decomposed-C mol^-1^	354 ± 107^b^	1541 ± 72^a^	246 ± 24^b^	0.000^∗∗∗^
LAC	pg decomposed-C mol^-1^	31 ± 8^a^	48 ± 23^a^	20 ± 6^a^	0.143


*Significant differences among the litters were tested through one-way ANOVA (*n* = 9, significance level ^∗∗∗^*P < 0.001*). Different letters within rows indicate significant differences among the litters (Tukey’s HSD test, α = 0.05).*

### Relationships between the Fungus and Its Substrate Quality

Stepwise regressions showed that the initial composition of the litter explained most of the variation in the metabolic responses of decomposition by *P. chrysosporium* after 14 days (*R*^2^ ≥ 0.97, *P* < 0.001, **Table 3**). The CUE and *q*CO_2_ were best predicted by the indexes of the soluble pools, i.e., the SOL, DOC, and DOC:DON ratio. The CUE was positively related to the DOC and negatively related to the DOC:DON ratio and SOL, whereas the opposite was observed for the *q*CO_2_. The stepwise regressions predicted that the CUE was high when the DOC was also high and when both the DOC:DON ratio and SOL were low. The fungal C accumulation C_f_ at day 14 was best predicted by the cell-wall composition, particularly the contents of Glu, Xyl, and KL.

**Table 3:** Stepwise regression equations relating the metabolic responses of *P. chrysosporium* after 14 days of maize litter decomposition to the initial chemical composition of the litters.

Dependent variable	Best regression equation	*R*^2^	*F*-value	*P*-value
C-use efficiency (CUE)	$0.223+0.141\,\;DOC-0.005\frac{DOC}{DON}-0.015\,\;SOL$	0.970	54.54	<0.001
Fungal C (C_f_)	1290.3+12.7⁢ Glu−41.1⁢ Xyl−58.1⁢ KL	0.994	289.1	<0.001
Metabolic quotient (*q*CO_2_)	$93.2+6.5\,\;SOL+2.1\frac{DOC}{DON}-43.9\,\;DOC$	0.971	54.9	<0.001


*DOC, dissolved organic C; DON, dissolved organic N; Glu, glucose; Xyl, xylose; KL, Klason lignin; SOL, soluble fraction; *n* = 9.*

The growth of *P. chrysosporium* also impacted the substrate chemical composition. A marked decrease in DOC was observed for all of the litter types during the first 14 days, and although the DOC content remained steady thereafter in the stem and root treatments, it reached a second peak at 56 days in the leaves (data not shown). The cell-wall contents decreased with decomposition for all of the litters. The impact of *P. chrysosporium* growth was most substantial on the leaves and led to a significant decrease in Glu, Xyl, Ara, Gal, and KL (**Supplementary Table 1**). Interestingly, the SOL content remained steady over the leaf and root decomposition and significantly decreased in the stem treatment.

**FIGURE 6:**
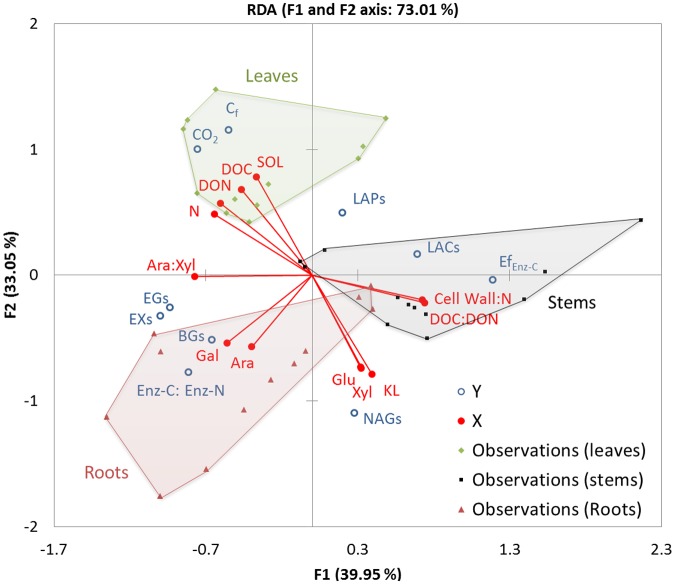
**Ordination plots of the first two axes of a redundancy analysis (RDA) of the metabolic and enzymatic responses (dependent variables) of *P. chrysosporium* during the decomposition of maize litters in relation to their chemical composition (independent variables).** The RDA was performed on center-reduced variables. The dependent variables are ordinated as open (blue) circles and independent variables in (red) lines terminated by full circles. The observations from the maize leaf, stem and root litters are shown by (green) diamonds, (black) squares and (red) triangles, respectively; the area covered by each litter treatments is colored (in green for leaves, gray for stems, red for roots). The dependent variables are the following: cumulative C mineralized (CO_2_) and C recovered in fungus (C_f_) in mg C g^-1^ initial litter C; specific enzyme activities in μmol min^-1^ mg^-1^ fungal C: βGs, EGs, EXs, LAPs, NAGs, LACs; ratios of enzymatic C to N acquisition activities represented by natural logarithms of (βG + EX + EG) to (LAP + NAG) activities (Enz-C: Enz-N); efficiency of enzymatic C decomposition for C-hydrolases in μg decomposed C mol^-1^ (Ef_Enz-C_). Independent variables are in (% initial litter C): dissolved organic C (DOC), dissolved organic N (DON), soluble fraction (SOL), N, arabinose (Ara), glucose (Glu), xylose (Xyl), galactose (Gal), Klason lignin (KL), Ara-to-Xyl ratio (Ara:Xyl), KL-to-N ratio (KL:N), DOC-to-DON ratio (DOC:DON), and cell wall-to-N ratio (Cell wall:N).

In the RDA ordination figure, axes 1 and 2 represent 39.95 and 33.05% of the variance in the metabolic and enzymatic responses of *P. chrysosporium* explained by the litter chemical composition over decomposition, respectively (**Figure 6**). The observed values for the three litter types varied and showed significant differences between their loadings (*P* < 0.001). Axis 1 was mainly explained by the DOC:DON and cell wall:N ratios, which were associated with stem observations on the right and with N, DON, and Ara:Xyl ratios, which were associated with leaf and root observations on the left. Axis 2 was mainly explained by the SOL, which was associated with leaf observations on the bottom and with cell-wall sugars (Glu, Ara, Gal, Xyl) as well as KL associated with root observations on the top, with stem observations at the center of this gradient. Among the response variables, mineralization (CO_2_) and fungal C accumulation (C_f_) were highly positively correlated with SOL and DON. These indexes of litter C consumption were negatively correlated with the cell-wall constituents and ran in the opposite direction to the NAG-specific activity, which likely reveals high levels of fungal material recycling activity. Specific C-acquiring hydrolase activities (βGs, EXs, EGs) and (βG + EX + EG):(LAP + NAG) ratios were correlated with Gal and the Ara:Xyl ratio, indicating that the composition of hemicellulose sugars was the main marker of C limitation in this experiment. The opposite direction indicated the enzymatic efficiency, specific oxidative activity (LACs) and N-acquiring activity (LAPs), and these response variables showed strong negative correlations with Gal and the Ara:Xyl ratio.

## Discussion

Despite being crucial for understanding how plant litter is transformed into microbial products, to our knowledge, the metabolic and enzymatic responses of litter decomposers have never been simultaneously quantified at the population level (Geyer et al., 2016). Further, as shown by Cotrufo et al. (2015), microbial products likely provide a greater contribution to C stabilization in soil than do the original plant litter compounds. Using a model microorganism growing on litter, this work balanced the key extracellular enzyme activities with the size of the microbial C pool as a function of time and substrate availability. Accordingly, we were able to introduce the concept of microbial investment in enzyme activities. Our results unambiguously showed that the contrasting chemical composition of the three investigated substrates induced distinct enzymatic strategies (combining investments and efficiencies) that resulted in different microbial C production and respiration. Our experimental approach offers the advantage of avoiding enzyme interference with soil mineral and organic constituents (Maire et al., 2013).

### Litter Decomposition by the Fungus

#### Substrate Degradation and Enzymatic Activities

*Phanerochaete chrysosporium* induced cumulative C mineralization was lowest in the roots (124 mg C–CO2 g^-1^ initial litter C), intermediate in the stems (127 mg C–CO2 g^-1^ initial litter C), and highest in the leaves (270 mg C–CO2 g^-1^ initial litter C) after 126 days. These numbers are higher than those reported in the few studies that have measured CO_2_ respiration during fungal growth on lignocellulosic substrates (Lekkerkerk et al., 1990; Allison et al., 2009). It is worth noting that the extent of the litter C mineralization was half of that reported for the same maize litter incorporated in soil (Machinet et al., 2009; Amin et al., 2014). Although simplified, this approach has the potential to mirror degradation processes in which soil-litter contact is reduced, such as in forests or no-till agricultural systems with mulch formation (Coppens et al., 2006).

*Phanerochaete chrysosporium* consumed large proportions of DOC and DON during growth on all of the maize litters, and this directly influenced the C metabolism. The SOL fraction was consumed on the stems and remained steady during decomposition on the leaves, which was likely caused by the concomitant replenishment of this fraction along with the degradation products and its consumption during decomposition (Moorhead et al., 2014). In the stem and root treatments, the degradation of structural cell-wall compounds was lower than that in the leaf treatment. The decreased lignin content in the leaves was consistent with values reported in the literature (e.g., 18–24% of the initial content of *Miscanthus sinensis* leaves for seven basidiomycetes; Osono, 2010). The response of the fungi to the initial litter quality was consistent with observations at the scale of the soil heterotrophic community, showing that litters with high soluble contents decomposed faster than litters with high lignin contents.

The degradation of the chemical components in the litters matched the succession in extracellular enzyme activities targeting these components and followed classically observed patterns (Šnajdr et al., 2011; Amin et al., 2014; Fanin et al., 2016). *P. chrysosporium* exhibited enzyme activities on the same order of magnitude as those reported for other litter-decomposing basidiomycetes (Voříšková et al., 2011; Talbot et al., 2015). The activities were already high after 14 days of decomposition for βG, EX, and LAC, and these enzymes likely acted in cooperation (synergetic effect) to more efficiently depolymerize the cell-wall network (Hu et al., 2011; Amin et al., 2014). EG was produced later once the cellulose fibrils were disconnected from the non-cellulosic matrix and thus more accessible. LAP synthesis was concomitant with fungal production and indicated fungal N needs, whereas NAG synthesis appeared later and was involved in the autolysis of senescing or dead mycelium for recycling N (Ray et al., 2012). The increased LAC activities in the last stage of decomposition for the leaves could have been related to the degradation of lignin, which began to be energetically beneficial for the fungus at this point. Even if lignin decay does not provide an energy gain, it provides access to other biochemically shielded resources, including energy-rich carbohydrates and proteins (Moorhead et al., 2013a).

#### Fungal Production and Metabolism

We assessed the fungal production by measuring the ergosterol concentrations in the litters as well as by using the ergosterol-to-fungal biomass conversion factor determined for *P. chrysosporium*. This conversion factor (1411 μg ergosterol g^-1^ fungal DM) is close to the mean ratio of 1716 μg ergosterol g^-1^ fungal DM proposed by Niemenmaa et al. (2008) for this fungus, and it is also in the range of values estimated by Voříšková et al. (2011) for *H. fasciculare* (1364 μg ergosterol g^-1^ fungal DM). Although, we are confident in the estimate of this factor, which was determined in liquid enriched medium, we cannot exclude the possibility that ergosterol may have been incorporated by the fungus in its membrane at higher proportions when grown on the litter substrates (Charcosset and Chauvet, 2001), which may have led to an overestimation of the fungal biomass. The C and N concentrations in *P. chrysosporium* were similar to those measured for this fungus grown on wood debris (Lekkerkerk et al., 1990), and the C: N ratio of 7.1 is consistent with the mean C:N measured for basidiomycete strains grown on grassland leaf litters (Mouginot et al., 2014). Similarly, the CUE values measured in this experiment (0.40–0.60) were in the range found for fungi in laboratory conditions by Keiblinger et al. (2010). The CUE values determined at the population scale were in the same range (0.40–0.68) as those shown by Geyer et al. (2016) at the community scale using similar methods (see Eq. 1). Except for the effect of community dynamics, which cannot be integrated into our approach, other drivers that affect CUE, such as substrate availability, have effectively been captured. By using the measured values of fungal C:N and the ergosterol-to-fungal biomass conversion factor, we calculated that the amounts of N accumulated in the fungal biomass would represent more than 100% of the initial litter N for the leaf and stem treatments and 61% for the root treatment on day 14. This unbalanced N calculation suggests an overestimation of the fungal biomass as mentioned earlier. It is also possible that the C:N ratio of the fungus was variable, with less N per unit of C incorporated during biosynthesis. Although changes in stoichiometry are often associated with shifts in microbial communities, stoichiometry at the scale of the individual microorganism species has also been observed when grown under different nutrient regimes in the laboratory (Vrede et al., 2002). Assuming, for example, that the fungal C:N ratio had increased by 10 after 14 days, then 78% (leaves), 89% (stems), and 44% (roots) of the initial litter N would have been incorporated into the fungus.

### Enzymatic Strategies of the Fungus

When reporting per unit fungal C, the enzyme activities defined the fungal investments in the different classes of enzymes. The investments in C-acquiring or N-acquiring hydrolases reported here represent the sum of the C or N enzymes, respectively, although the results were the same when considering the enzyme activities separately (not shown). Indeed, the fungus modulated its investments as a function of the substrate quality. The lowest investment in enzyme activities was found in the stem treatment regardless of the type of enzyme. We believe that enzyme synthesis itself requires an investment of microbial resources, particularly N (Mooshammer et al., 2014); therefore, the N-limiting conditions observed in the stems may have limited the ability of *P. chrysosporium* to invest in enzymes. The protein C:N ratio is usually much lower than the microbial biomass C:N (Moorhead et al., 2012). Additionally, enzyme synthesis may only be beneficial if the targeted substrates are available in concentrations high enough to offset the cost of synthesis (Allison et al., 2014). In the root treatment, *P. chrysosporium* showed the highest investment in C-acquiring hydrolytic and oxidative activities, which indicates a limitation of C and a need for an energy supply; this result is consistent with the framework of microbial allocation patterns, which predict an increased allocation to enzymes that target the limited element (Moorhead et al., 2012; Averill, 2014). However, the fungal investment in N-acquiring hydrolase activities was the highest for leaves, which may be explained by a strong N demand to support fungal production on this litter. As previously mentioned, the present results demonstrate that enzyme synthesis was inducible and responsive to differences in substrate quality (Moorhead et al., 2013b) and stoichiometric needs (Sinsabaugh et al., 2008).

The most striking result is that the highest enzyme efficiency was found in the stem treatment despite *P. chrysosporium* showing a lower investment toward enzyme activities in the stems relative to the leaf and root treatments. The lowest enzyme efficiencies in the root treatment were compensated for by the highest investments in enzyme activities. The relationships between the enzyme efficiencies and the chemical recalcitrance of the substrate remain unclear (Sinsabaugh et al., 2002; Wickings et al., 2012; Amin et al., 2014; Fanin et al., 2016). In the present work, differences in the enzyme efficiencies observed between the stems and roots could not be explained by differences in the lignin content, which was similar for the two litters. The enzyme efficiencies were inversely correlated with the Ara:Xyl ratio and Gal content as shown by the RDA. This result suggests that when hemicelluloses were more enriched in Ara and Gal, the efficiency of the enzymes was lower. Hemicelluloses are composed of Xyl chains branched with side chains of mainly Gal and Ara residues, thereby exhibiting a ramified structure that hampers their degradation and favors interconnections with lignin (Bertrand et al., 2006; Hu et al., 2011). The high number of linkages between polymers and the heterogeneity of these linkages in the plant cell walls, which was observed in the root and leaf litters, produced cell-wall polymers that were less accessible for enzymes and therefore more difficult to depolymerize, thereby resulting in the lower efficiency of enzyme activities.

### Metabolic Responses of the Fungus

Carbon use efficiency provides a synthetic picture of the fate of C that is metabolized by decomposers, which is either respired (during microbial biosynthesis, maintenance, extracellular enzyme synthesis, and overflow mechanisms) or incorporated into new microbial biomass (Manzoni et al., 2012). As stated by Geyer et al. (2016), the CUE determined at the timescale used in this work likely captured the utilization of the substrate before significant biomass turnover. Under this rationale, the findings of this study provide some insights that help us understand C cycling at the scale of an individual species population and, to some extent, at the scale of the microbial community because our experimental design captured drivers at these ecological scales. Our regression results showed that the CUE was highly dependent on the content of the soluble compounds and the stoichiometry of the soluble fraction. The metabolic quotient (*q*CO_2_) was inversely related to the same variables. The soluble sugars and other easily assimilated compounds of the litters serve as the initial fuel for microorganisms and provide resources that can be allocated toward enzyme synthesis. Enzymatic strategies then explain how the microorganisms maintain their supply from litter structural components by replenishing the soluble faction with degradation products, which was observed for the leaves. The leaf treatments induced the highest fungal biosynthesis throughout the experiment and the lowest *q*CO_2_, indicating low maintenance energy requirements (Allison, 2014; Mooshammer et al., 2014) and moderate respiration costs for enzyme synthesis. These results also demonstrate the ability of the microorganisms to obtain and assimilate new available products from enzyme activities. Inversely, the stem treatments induced the lowest fungal biosynthesis and the highest *q*CO_2_. In a recent analysis of the literature, Spohn (2015) observed that microorganisms respired more C per unit of microbial C when the substrate N content was low. Our results support two of the explanations considered: (i) N mining mechanisms, whereby microbes use available C to gain energy to acquire N embedded in recalcitrant polymers, and (ii) overflow respiration, whereby excessive C is excreted. A shift in metabolic pathways that require higher maintenance costs may also explain the high *q*CO_2_ observed in the stem treatments. In addition to the high respiration costs, we believe that fungal biosynthesis was limited because the fungus did not have sufficient available degraded compounds, which was a direct consequence of its enzyme strategy and substrate recalcitrance. The low investment in enzyme activities because of the N-limiting conditions was not fully compensated for by the high efficiency in the stem litter, which resulted in a low CUE for the stems. For the roots, the CUE had intermediary values that were low relative to that of the leaves. Because the fungus exhibited high investments in enzyme activities in the root treatment at a low efficiency, we can assume that C was more difficult to access, which both limited the mycelium production and raised the respiration costs of extracellular enzyme synthesis. Finally, we believe that for the leaf litter, the fungus moderately invested in the enzyme activities because the efficiency was relatively high, and this led to effective plant cell-wall degradation and resulted in a high biomass production rate and moderate respiration costs, thereby yielding the highest CUE.

## Conclusion

Although we expected a strong correlation between the enzyme efficiency and CUE of the fungus *P. chrysosporium* when grown on maize root, stem, or leaf litters, the CUE was initially dependent on the initial availability and/or the replenishment of the soluble fraction of substrates along with the degradation products. The fungal production strategies relied on both the fungal ability to invest in enzyme activities and the efficiency of these enzyme activities. A low investment in enzyme activities or low enzyme efficiency was found to limit the CUE. These results are important for modeling microbial allocation strategies in enzyme-based decomposition models (Cotrufo et al., 2013; Moorhead et al., 2013b). This study provides high-resolution data that are required to constrain and parameterize such fine-scale models. The results also emphasize the potential constraints to enzyme investments due to nutrient limitation, or to enzyme efficiency due to the chemical recalcitrance and high interconnection of the polymers in the plant cell wall, should be represented in models. They further illustrate that the CUE should not be a fixed parameter in models because it can be highly variable, even for a single-species microorganism. Instead, the CUE should vary as a result of microbial activity and substrate composition controls. This experiment represents one of the first steps in more precisely determining the cost-benefit relationships of microbial investments in extracellular enzymes.

## Author Contributions

GL, AG-C, SR, and IB conceived the project. GL, AG-C, and IB designed the experiment. GL and AG-C performed the experiment. GL, SR, and IB interpreted the data. GL, AG-C, SR, and IB wrote the manuscript.

## Conflict of Interest Statement

The authors declare that the research was conducted in the absence of any commercial or financial relationships that could be construed as a potential conflict of interest.
