# Supplementary material for: Enzymatic Strategies and Carbon Use Efficiency of a Litter-Decomposing Fungus Grown on Maize Leaves, Stems, and Roots
Source: Front Microbiol. 2016 Aug 26;7:1315. doi: 10.3389/fmicb.2016.01315 (PMC4999447; doi:10.3389/fmicb.2016.01315)
Supplement: Supplementary file 1 [file Table_1.DOCX]

**Supplemental information**

**Table 1.** C mineralization and chemical composition of the maize litter after 126 days of incubation without (control) and with *Phanerochaete chrysosporium*

| % initial C | Control litter | | | | | | | | | | | | | |  | Litter + *P. chrysosporium* | | | | | | | | | | | | | | | | |
| --- | --- | --- | --- | --- | --- | --- | --- | --- | --- | --- | --- | --- | --- | --- | --- | --- | --- | --- | --- | --- | --- | --- | --- | --- | --- | --- | --- | --- | --- | --- | --- | --- |
|  | Leaves | | |  |  | Stems | | |  |  | Roots | | |  |  | Leaves | | |  |  | Stems | | |  |  | Roots | | |  |  | *P*-value | |
| C mineralized | 0.6 | ± | 0.1 | ^c^ |  | 0.1 | ± | 0.0 | ^c^ |  | 0.2 | ± | 0.0 | ^c^ |  | 28.6 | ± | 1.9 | **^a^** |  | 13.5 | ± | 0.6 | ^b^ |  | 12.9 | ± | 1.2 | **^b^** |  | <0.0001 | ^***^ |
| Dissolved organic C | 7.2 | ± | 0.3 | ^a^ |  | 5.6 | ± | 0.1 | ^b^ |  | 4.1 | ± | 1.0 | ^c^ |  | 4.8 | ± | 0.1 | **^bc^** |  | 2.4 | ± | 0.1 | **^d^** |  | 2.6 | ± | 0.1 | **^d^** |  | <0.0001 | ^***^ |
| Dissolved organic N^e^ | 0.4 | ± | 0.0 | ^a^ |  | 0.1 | ± | 0.0 | ^cd^ |  | 0.2 | ± | 0.0 | ^b^ |  | 0.4 | ± | 0.0 | ^a^ |  | 0.1 | ± | 0.0 | ^d^ |  | 0.2 | ± | 0.0 | ^bc^ |  | <0.0001 | ^***^ |
| Soluble fraction | 33.1 | ± | 2.8 | ^a^ |  | 21.2 | ± | 0.9 | ^b^ |  | 16.6 | ± | 3.3 | ^bc^ |  | 34.9 | ± | 0.2 | ^a^ |  | 14.1 | ± | 0.6 | **^c^** |  | 14.3 | ± | 0.7 | ^c^ |  | <0.0001 | ^***^ |
| Cell Wall to N ratio | 40.5 | ± | 2.9 | ^c^ |  | 284.0 | ± | 10.7 | ^a^ |  | 111.9 | ± | 15.7 | ^b^ |  | 37.9 | ± | 5.3 | ^c^ |  | 283.4 | ± | 5.3 | ^a^ |  | 105.7 | ± | 3.9 | ^b^ |  | <0.0001 | ^***^ |
| Total cell wall sugars | 49.8 | ± | 7.1 | ^a^ |  | 57.4 | ± | 0.4 | ^a^ |  | 57.9 | ± | 6.0 | ^a^ |  | 22.8 | ± | 0.9 | **^b^** |  | 48.5 | ± | 7.4 | ^a^ |  | 57.7 | ± | 1.3 | ^a^ |  | 0.0001 | ^***^ |
| Glucose | 29.9 | ± | 4.0 | ^a^ |  | 37.6 | ± | 0.1 | ^a^ |  | 36.2 | ± | 3.7 | ^a^ |  | 13.5 | ± | 0.5 | **^b^** |  | 31.0 | ± | 4.8 | ^a^ |  | 34.6 | ± | 1.2 | ^a^ |  | 0.0001 | ^***^ |
| Xylose | 15.8 | ± | 2.4 | ^a^ |  | 17.1 | ± | 0.5 | ^a^ |  | 17.0 | ± | 1.8 | ^a^ |  | 7.3 | ± | 0.5 | **^b^** |  | 14.8 | ± | 1.8 | ^a^ |  | 18.0 | ± | 1.1 | ^a^ |  | 0.0001 | ^***^ |
| Arabinose | 2.7 | ± | 0.5 | ^ab^ |  | 1.9 | ± | 0.0 | ^bc^ |  | 2.9 | ± | 0.3 | ^a^ |  | 1.1 | ± | 0.1 | **^c^** |  | 1.5 | ± | 0.2 | ^bc^ |  | 3.0 | ± | 0.3 | ^a^ |  | <0.0001 | ^***^ |
| Galactose | 0.8 | ± | 0.2 | ^b^ |  | 0.5 | ± | 0.0 | ^b^ |  | 1.1 | ± | 0.1 | ^a^ |  | 0.4 | ± | 0.0 | ^b^ |  | 0.4 | ± | 0.1 | ^b^ |  | 1.3 | ± | 0.2 | ^a^ |  | <0.0001 | ^***^ |
| Arabinose to Xylose ratio | 0.17 | ± | 0.00 | ^a^ |  | 0.11 | ± | 0.01 | ^bc^ |  | 0.17 | ± | 0.00 | ^a^ |  | 0.16 | ± | 0.01 | ^ab^ | | 0.10 | ± | 0.00 | ^c^ |  | 0.17 | ± | 0.03 | ^a^ |  | 0.0047 | ^**^ |
| Klason lignin | 9.8 | ± | 0.5 | ^b^ |  | 15.1 | ± | 0.4 | ^a^ |  | 15.6 | ± | 0.8 | ^a^ |  | 6.1 | ± | 0.4 | **^c^** |  | 15.1 | ± | 0.4 | ^a^ |  | 15.1 | ± | 0.2 | ^a^ |  | <0.0001 | ^***^ |
| Klason lignin to N ratio | 6.0 | ± | 0.4 | ^c^ |  | 54.5 | ± | 1.1 | ^a^ |  | 18.9 | ± | 3.1 | ^b^ |  | 4.3 | ± | 0.3 | ^c^ |  | 54.0 | ± | 2.2 | ^a^ |  | 20.2 | ± | 0.8 | ^b^ |  | <0.0001 | ^***^ |
| Lignocellulose index | 0.17 | ± | 0.03 | ^a^ |  | 0.21 | ± | 0.00 | ^a^ |  | 0.21 | ± | 0.01 | ^a^ |  | 0.21 | ± | 0.01 | ^a^ |  | 0.24 | ± | 0.02 | ^a^ |  | 0.21 | ± | 0.00 | ^a^ |  | 0.0720 |  |

Values represent the means of the replicated microcosms ± standard errors (n = 2 for the control litter, n = 3 for the litter inoculated with *P. chrysosporium).* Significant differences among the litters were tested using a one-way ANOVA (n = 15, significance levels *^**^P < 0.01, ^***^P<0.001*; P-values corrected by the Benjamini and Hochberg procedure). Different letters within rows indicate significant differences among the litters (Tukey's HSD test, α = 0.05).

Bold letters represent significant differences between the treatment with *P. chrysosporium* and the control.

^e^ % initial dry matter
